# Le kyste de la vallécule symptomatique chez l’adulte: à propos de 4 cas

**DOI:** 10.11604/pamj.2018.31.36.16333

**Published:** 2018-09-18

**Authors:** Mehdi Hasnaoui, Mohamed Masmoudi, Jamel Chefai, Nouha Ben Hmida, Khalifa Mighri, Nabil Driss

**Affiliations:** 1 Service d'Oto-Rhino-Laryngologie et Chirurgie Cervico-Faciale, CHU Tahar Sfar, Mahdia, Tunisie

**Keywords:** Kyste de la vallécule, laryngoscopie direct, résection endoscopique, marsupialisation, Vallecular cyst, direct laryngoscopy, endoscopic resection, marsupialisation

## Abstract

Le kyste de la vallécule est une lésion bénigne rare. Ces kystes sont souvent asymptomatiques chez l'adulte. Les auteurs rapportent 4 cas de kystes de la vallécule symptomatique de l'adulte. Trois patients ont consulté pour une dysphagie haute mixte associée à une dysphonie. Le quatrième patient se plaignait d'une sensation de corps étranger dans la gorge. La laryngoscopie a montré une formation kystique dans la région valléculaire gauche dans tous les cas. La tomodensitométrie a confirmé la présence d'une formation kystique de la vallécule. Le traitement a consisté en une marsupialisation du kyste dans deux cas et une résection endoscopique dans deux cas. À partir de ces 4 cas cliniques, nous nous proposons de préciser les particularités diagnostiques et thérapeutiques de cette affection.

## Abstract (en)

Symptomatic vallecular cyst is a rare benign lesion. These cysts are often asymptomatic in the adult. We here report 4 cases of symptomatic vallecular cyst in the adult. Three patients presented with mixed high dysphagia associated with dysphonia. The fourth patient complained of foreign body sensation in the throat. Laryngoscopy showed a cyst in left vallecular region in all cases. CT scan confirmed vallecular cyst. Treatment was based on marsupialisation of the cyst in two cases and endoscopic resection in two cases. This study suggests to clarify the diagnostic and therapeutic features of this disorder.

## Introduction

Le kyste de la vallécule est une lésion bénigne rare développée aux dépens de glandes muqueuses de la vallécule. L'incidence exacte du kyste valléculaire est inconnue [1, 2]. Chez l'adulte, les kystes valléculaires sont souvent asymptomatiques, pour deux raisons; d'une part à cause de leur petite taille, d'autre part à cause d'une filière pharyngolaryngée assez large [3, 4]. À partir de ces 4 cas cliniques, nous nous proposons de préciser les particularités diagnostiques et thérapeutiques de cette affection.

## Méthodes

Il s'agit d'une étude rétrospective de 4 cas de kyste de la vallécule colligés entre les années 1998 et 2016. L'étude des dossiers a été faite à l'aide d'une fiche d'exploitation qui a per¬mis de recueillir les données épidémiologiques, cliniques, paracliniques et évolutives. Le diagnostic était retenu dans tous les cas sur les don¬nées de l'endoscopie, du scanner et l'examen anatomopathologique de la pièce opératoire.

## Résultats

**Figure 1:**
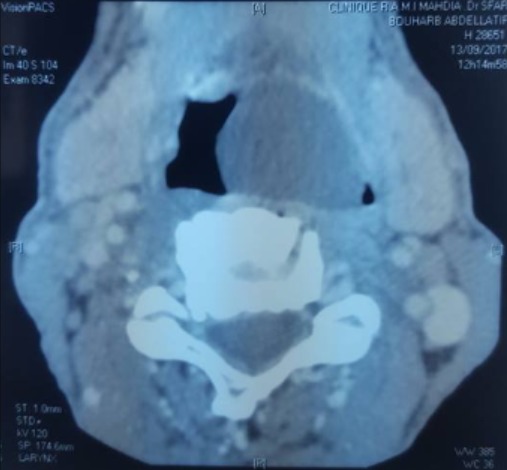
Scanner cervical en coupe axiale montant une masse kystique comblant en partie l’oropharynx

**Figure 2:**
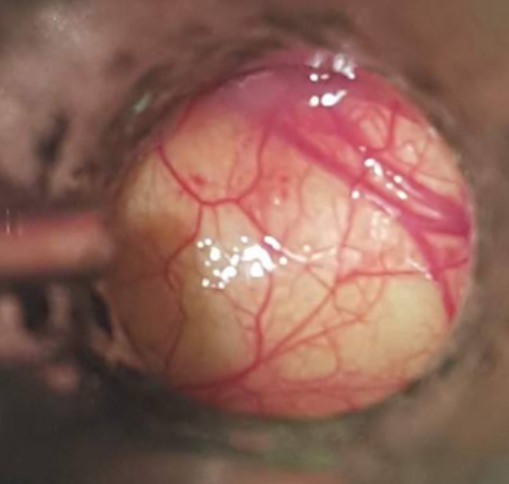
Vue endoscopique de la masse kystique de la vallécule

**Figure 3:**
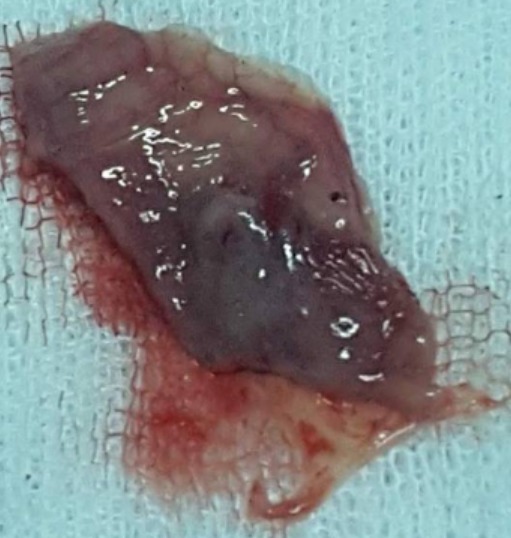
Pièce opératoire de marsupialisation d’un kyste de la vallécule

Il s'agissait de quatre hommes d'âge moyen 56 ans (46 à 63 ans). Il n'y avait pas des antécédents pathologiques particuliers. Le délai moyen du diagnostic était de six mois avec des extrêmes allant de 2 mois à 12 mois. Trois patients ont consulté pour une dysphagie haute mixte associée à une dysphonie. Le quatrième patient se plaignait d'une sensation de corps étranger dans la gorge sans d'autres signes associés. Aucun patient n'avait une dyspnée (Tableau 1). La laryngoscopie a montré une formation kystique dans la région valléculaire gauche. La tomodensitométrie cervicale a mis en évidence une formation kystique uniloculaire bien limitée, hypodense et occupant l'étage sus-glottique. Le diamètre moyen de cette formation kystique était de 2,5 cm avec des extrêmes allant de 2 cm à 3,5cm (Figure 1). Sous anesthésie générale, tous les patients ont été intubés afin d'effectuer une laryngoscopie directe. Celle-ci a montré une formation kystique jaunâtre, à surface lisse, comblant la vallécule gauche (Figure 2). Dans un cas, le kyste était volumineux, faisant 3,5 cm de grand axe et obscurcissant la vue du larynx, ce qui a rendu l'intubation difficile. Dans les autres cas, l'intubation était relativement facile. Nous n'avons pas eu recours à la ponction du kyste ni à la trachéotomie. Deux patients ont eu une marsupialisation du kyste avec les micro-instruments laryngés, après aspiration se son contenu (Figure 3). Dans les deux autres cas, le traitement a consisté en une dissection du kyste par des micro-instruments laryngés suivie de son exérèse subtotale. L'examen anatomopathologique définitif a conclu à un kyste valléculaire bénin revêtu d'une muqueuse de type respiratoire. L'évolution était bonne dans tous les cas. Il n'y avait pas de récidive, après un recul moyen de 14 mois.

**Tableau 1:** Caractéristiques cliniques et para-cliniques des 4 patients

	Patient 1	Patient 2	Patient 3	Patient 4
Age	46 ans	59 ans	63 ans	56 ans
Sexe	Masculin	Masculin	Masculin	Masculin
Motif de consultation	Dysphonie Dysphagie	Dysphonie Dysphagie	Dysphonie Dysphagie	Sensation de corps étranger
Délai de consultation	6 mois	4 mois	12 mois	2 mois
Laryngoscopie	Formation kystique de vallécule Gche	Formation kystique de vallécule Gche	Formation kystique de vallécule Gche	Formation kystique de vallécule Gche
TDM	Formation kystique uniloculaire hypodense de 35 x 30mm	Formation kystique uniloculaire hypodense de 25 x 20mm	Formation kystique uniloculaire hypodense de 20 x 15mm	Formation kystique uniloculaire hypodense de 20 x 15mm

## Discussion

Le kyste de la vallécule est une pathologie rare. Il est de type rétentionnel. L'inflammation chronique, aboutissant à l'obstruction des glandes à mucus en serait la cause principale. Toutefois, une étiopathogénie congénitale a également été envisagée car certains kystes valléculaires ont été décrits chez des nouveau-nés [1, 3, 5, 6]. Le kyste de la vallécule survient à tout âge mais il est moins fréquent chez les enfants que chez les adultes [6, 7]. Les symptômes varient selon l'âge et la taille du kyste. Chez l'enfant le kyste de la vallécule est potentiellement mortel causant une obstruction des voies respiratoires. Dans ce cas le kyste peut se révéler par un stridor et une détresse respiratoire. Ceci est expliqué par l'étroitesse de la filière laryngée chez l'enfant [5, 6, 8]. Par contre, chez l'adulte, les symptômes sont moins dangereux et peuvent se manifester par une dysphagie, des paresthésies pharyngées à type de sensation de corps étranger ou une dysphonie. Parfois, le kyste de la vallécule est asymptomatique et découvert lors d'un examen de routine du larynx, d'une intubation ou d'un examen radiologique notamment le scanner et l'imagerie par résonance magnétique [3-6]. Trois de nos patients ont consulté pour une dysphagie associée à une dysphonie sans dyspnée. Le quatrième patient avait uniquement une sensation de corps étranger dans la gorge. Ce qui est remarquable dans cette série, c'est que nous avons deux cas de kyste de vallecule relativement volumineux alors que la symptomatologie et le tableau clinique n'étaient pas franchement graves.

L'examen fibroscopique est pratiqué de première intention. Le diagnostic est, ensuite, posé par l'endoscopie laryngée sous anesthésie générale qui retrouve, dans une vallécule une tuméfaction claire, régulière, dont la surface est parcourue par un fin lacis vasculaire. Sur le plan radiologique, l'échographie ou la TDM peuvent facilement mettre en évidence la nature kystique de la lésion. Le scanner demeure toutefois l'examen essentiel en objectivant une néoformation sus-glottique régulière, homogène, hypodense, ne prenant pas le contraste [2, 5, 6]. L'imagerie par résonance magnétique peut également être pratiquée et montre une lésion homogène en hyposignal T1 et hypersignal T2. Cependant, elle ne semble pas apporter d'éléments supérieurs à ceux du scanner, qui devra rester l'examen de première intention [2, 5]. Le traitement repose sur la chirurgie. Celle-ci peut se faire par voie externe ou endoscopique. L'abord cervical externe augmente la durée de l'anesthésie et de l'hospitalisation avec un risque de blessure de nerf laryngé supérieur et de cicatrice cervicale. Cet abord est rarement utilisé [5]. Dans les cas des kystes volumineux, nous n'avons pas recours à la trachéotomie ni à l'aspiration de contenu du kyste afin de faciliter l'exposition de la glotte ni à l'intubation fibroscopique vigile comme ca était décrit dans la littérature par la plupart des auteurs. L'intubation a été faite selon la méthode classique. Lors d'un traitement endoscopique, plusieurs attitudes peuvent être adoptées: une incision simple du kyste avec aspiration de son contenu, cette technique expose à une plus grande fréquence de récidives; une marsupialisation aux micro-instruments laryngés, au microdebrideur, au laser CO2 ou encore par électrocoagulation; et enfin, l'exérèse complète du kyste [3-5, 9]. Chez nos patients, nous avons réalisé la marsupialisation et l'exérèse du kyste à l'aide des micro-instruments laryngés. Nous avons eu recours à la marsupialisation lorsque l'exérèse complète du kyste a été jugée difficile. Avec cette technique, il n'y avait pas de complications. Le saignement per-opératoire était minime et il a cédé spontanément et nous n'avons aucun cas de récidive.

## Conclusion

Le kyste valléculaire symptomatique de l'adulte est rare. Le diagnostic est porté par l'endoscopie laryngée. L'exérèse endoscopique aux micro-instruments laryngés, au microdebrideur, au laser CO2 ou encore par électrocoagulation est actuellement le traitement de choix. Il n'y a pas de différence significative entre ses différentes techniques. Le traitement endoscopique pose le problème de récidive de ces kystes d'où une surveillance régulière s'avère indispensable.

### Etat des connaissances actuelles sur le sujet

- Chez l'adulte, les kystes de vallécule sont souvent asymptomatiques, pour deux raisons: d'une part à cause de leur petite taille (souvent moins de 8 mm de diamètre), d'autre part à cause d'une filière pharyngo-laryngée assez large;
- Les Kystes laryngés, par leur localisation et volume, peuvent poser un problème d'intubation. Dans ce cas, la plupart des auteurs proposent d'affaisser le kyste par ponction directe et aspiration de son contenu afin de faciliter l'exposition de la glotte ou de réaliser une trachéotomie au préalable.

### Contribution de notre étude à la connaissance

- Tous nos cas de kyste de la vallécule sont symptomatiques vu que leurs tailles dépassent 2 cm;
- Dans les cas des kystes volumineux, nous n'avons pas recours à la trachéotomie ni à l'aspiration de contenu du kyste afin de faciliter l'exposition de la glotte ni à l'intubation fibroscopique vigile comme ca était décrit dans la littérature par la plupart des auteurs. L'intubation a été faite selon la méthode classique.

## Conflits d’intérêts

Les auteurs ne déclarent aucun conflit d'intérêts.

## Contributions des auteurs

Tous les auteurs ont contribué à la prise en charge de la patiente et à la rédaction du manuscrit. Tous les auteurs ont lu et approuvé la version finale du manuscrit.
